# Ultra-Processed Food in an Inpatient Mental Health Setting

**DOI:** 10.1192/bjo.2025.10516

**Published:** 2025-06-20

**Authors:** Anthony Onjewu, Ed Silva, Anthony Onjewu

**Affiliations:** Merseycare NHS Foundation Trust, Liverpool, United Kingdom

## Abstract

**Aims:** The consumption of ultra-processed food (UPF) is associated with many adverse health outcomes including cardiometabolic disorders, mental health disorders and mortality.

The aim of the service evaluation project is to assess the menu items of a 32-beded low secure forensic mental health hospital against the NOVA criteria for ultra-processed food. All the inpatients have a variety of severe mental health illnesses such as schizophrenia or bipolar. All are treated with antipsychotics and rates of complex physical health comorbidity are high.

**Methods:** 14 different menu items available from the catering department were analysed and the NOVA classification was assigned by reference to the ingredient list. The percentage of ultra-processed food in each menu item was calculated based on amount of NOVA 4 (ultra-processed food) items contained in relations to the total number of each food composition.

**Results:** Analysis of all 14 menu items, using the NOVA criteria, showed they contained about 68% of ultra-processed food material. This included unexpected items such as roast potatoes and omelettes. Each menu item was wrapped in plastic and had significant amount of processed material, artificial flavouring, colouring and other preservatives sufficient to be classed as ultra-processed food.

**Conclusion:** ‘Don’t just screen, intervene’ is the motto used to try and improve the physical health of people with severe mental illness. The Lester tool used to assess the cardiometabolic health of people with severe mental health disorder, focuses on the individual person but without the consideration of the institutional context that surrounds those detained in the forensic mental health unit for many years. The interventions all include advice to eat healthily which is impossible if all the food provided is ultra-processed. Whilst individual organisations might be able to change their catering standards to remove ultra-processed food from their menus, a systemic change to nutritional standards for mental health inpatients may be more effective.

